# Piezoelectric nanogenerator for bio-mechanical strain measurement

**DOI:** 10.3762/bjnano.13.14

**Published:** 2022-02-07

**Authors:** Zafar Javed, Lybah Rafiq, Muhammad Anwaar Nazeer, Saqib Siddiqui, Muhammad Babar Ramzan, Muhammad Qamar Khan, Muhammad Salman Naeem

**Affiliations:** 1 School of Arts and Design, National Textile University, 37610, Faisalabad, Pakistan; 2 School of Engineering and Technology, National Textile University, 37610, Faisalabad, Pakistan; 3 Sapphire Finishing Mills Limited, Lahore, Pakistan; 4 Department of Clothing, Faculty of Textile Engineering, National Textile University, Karachi Campus, Pakistan

**Keywords:** electrospinning, human body angle measurement, nanofibers, piezoelectric, PVDF

## Abstract

Piezoelectric materials have attracted more attention than other materials in the field of textiles. Piezoelectric materials offer advantages as transducers, sensors, and energy-harvesting devices. Commonly, ceramics and quartz are used in such applications. However, polymeric piezoelectric materials have the advantage that they can be converted into any shape and size. In smart textiles, polyvinylidene fluoride (PVDF) and other piezoelectric polymers are used in the form of fibers, filaments, and composites. In this research, PVDF nanofibers were developed and integrated onto a knitted fabric to fabricate a piezoelectric device for human body angle monitoring. Scanning electron microscopy and X-ray diffraction analyses were used to study the morphology and to confirm the beta phase in fibers. The results reveal that the nanofibers made from solutions with high concentration were smooth and defect-free, compared to the fibers obtained from solutions with low concentration, and possess high crystallinity as well. Under high dynamic strain more output voltage is generated than under low dynamic strain. The maximum current density shown by the device is 172.5 nA/cm^2^. The developed piezoelectric nanofiber sensor was then integrated into a knitted fabric through stitching to be used for angle measurement. With increasing bending angle, the output voltage increased. The promising results show that the textile-based piezoelectric sensor developed in this study has a great potential to be used as an angle measuring wearable device for the human body due to its high current density output and flexibility.

## Introduction

Smart textiles are normally elevated to value-added textile products with improved properties and characteristics [1]. They exhibit properties of a textile with some added characteristics. Smart textiles are obtaining by combining conventional textile techniques, such as knitting or weaving, with different technologies of electronics [2]. The world is moving towards intelligent or smart textiles. In 2012, the size of the smart textile market was almost $289.5 million and surged to $1,500 million in 2020. The integration of electronically active fibers or yarns in textile substrates is the basis of smart textiles [3]. Textile-based sensors and electrodes are composed of conductive fibers, threads, or fabrics [4]. Their use for physiological and medical examination has been rising rapidly in the last couple of years. Textile-based sensors, being flexible, are easy to fit in a garment and create no barrier to the wearer. Nowadays, wearable sensors based on conductive threads and conductive polymers are capable of measuring vital signs of the human body [4–5]. Tognetti et al. [6] designed and developed a resistive strain sensors for movement analysis. They integrated an electrically conductive elastomer into a fabric, which was then able to detect the posture and the movement of the human body. Retrieved data from these strain sensors were compared with conventional motion tracking systems. The results show promising performance for body posture classification and reconstruction. Similarly, for measuring human body angles, piezoresistive sensors were developed and characterized under bending and stretching regarding the application as strain sensors [7]. Knitted piezoresistive fabrics were used to develop sensors that were a wearable type of a goniometer. These sensors were then tested under static and dynamic conditions. For another application, researchers designed and developed a purely textile-based capacitive pressure sensor to be integrated and embedded into the garments to monitor and measure human body pressure. These sensors were beneficial for pressure sore prevention, rehabilitation, and the detection of movement during activities. Further, these sensors were comfortable and bendable and were applied onto the upper portion of an arm to detect the deflection of the forearm during muscle bending [8]. Park et al. [9] developed a self-powered piezoelectric sensor for monitoring the pulse rate in real time. A pressure sensor was attached to the epidermis for monitoring pulse and assessing personal health status. Traditional sensors for pulse monitoring can detect bio-signals of the human body but they have the limitation of power supply, which will restrict the operation of the wearable devices for medical purposes. Hence, piezoelectric sensors were used for monitoring bio-signals of the human body without limitation of power supply. Moreover, Lorussi and co-workers developed a smart textile garment by embedding a strain sensor into an ordinary garment. For the piezoelectric effect, the conductive blend was applied onto the fabric, which resulted in a change in resistance under strain. This phenomenon was used in gloves, car seats, and leotards for determining body posture, shape, and gesture. They primarily focused on studying a leg pad that was able to perceive the knee movement and posture [10]. Piezoelectric sensors have a wide range of applications including sidewalks or crosswalks that collect energy from vibrations, which can be store in batteries [11]. Moreover, piezoelectric sensors can be used at workplaces and gyms to collect energy from machine vibrations [12]. These sensors are embedded under the shoes so that the pressure exerted during walking or running can be converted into energy and can be used for different applications. Piezoelectric sensors can also be used under mats and floors so the pressure due to footsteps can be utilized as a source to generate energy [13]. Besides these, piezoelectric sensors can also be used for sensing human body motion and monitoring physical health parameters, such as electrocardiograms [14–19]. Polyvinylidene fluoride (PVDF), having a semi-crystalline structure, is generally synthesized through polymerization of vinylidene difluoride [20]. It generally has four crystalline phases, namely α, β, ε, and γ. Among them, the beta phase possesses the highest dipole movements, while the other phases are usually non-polar as their structural packing is anti-parallel [21]. Usually, PVDF is non-reactive towards acids and bases. It was discovered in 1969 that PVDF can produce electrical signals. Thus, it can be used in various applications of energy harvesting, in various forms such as fibers, films, monofilaments, and powder. This material is trending in textile-based research where different researchers are working to manufacture smart textiles to generate energy [22–23]. Nanofibers have many technical applications such as in air and liquid filtration [24–25], tissue engineering [26–27], drug delivery [28], wound dressings [29], sound adsorption [30], cosmetics [31], and sensor devices [32–34]. In filtration processes, electrospun nanofibers can be employed for removing volatile organic compounds (VOCs) from the atmosphere. To protect people from bacteria, viruses, smog, and dust, nanofibers are utilized in medical face masks. These masks will not allow the particles to be inhaled because of the small pore size of the nanofibrous scaffold, while oxygen molecules are small enough to pass through these pores. Nanofibers are also used in other medical applications, for instance, for developing artificial organs and blood vessels, and in gene and drug delivery [35]. Monitoring joint angles through wearable systems enables human posture and gesture to be reconstructed as a support for physical rehabilitation both in clinics and at the patients’ home [36]. To date, wearable sensors used for monitoring body movements in the market are battery-based. The battery needs to be worn all the time. Also, it needs to be charged or replaced, which makes its application impractical. In this work, we present a proof of concept for using a nanofibrous-based piezoelectric sensor composed of PVDF, which is capable of monitoring body angles. This sensor will be able to replace the battery being used in commonly available products and is more breathable, lightweight, and flexible. The developed sensor has been characterized through advanced techniques. The current density has been calculated and compared with the current state of the art. To the best of our knowledge, the PVDF-based nanofibrous device developed in this study is superior to previously reported ones.

## Experimental

### Materials

Polyvinylidene fluoride (PVDF) obtained from Alfa Aesar was used as a piezoelectric material. Dimethylformamide (DMF) and acetone from Sigma-Aldrich were used as solvents without any further purification. A conductive tape was used to make electrodes. Knitted fabric was used for the integration of the nanofibrous mesh for human body angle measurement.

### Fabrication of the nanofibrous mesh and its characterization

PVDF solutions with varying concentrations (12, 14, and 16 wt %) were prepared in an acetone/DMF mixture (1:2.3 by volume). PVDF was dissolved in the acetone/DMF mixture at 120 °C in a sealed container under stirring for 4 h followed by incubating the solutions at room temperature for 24 h before electrospinning. A conventional electrospinning process was used to create the piezoelectric electrospun nanofibers. The polymeric solution was pumped from a metallic syringe needle of 0.4 mm inner diameter at a flow rate of 3.5 mL/h. The fibers were collected on a stationary collector placed at a working distance of 15 cm. A constant voltage of 15 kV was used for all the experiments. The nanofibrous meshs were first dried at room temperature in the fume hood for 24 h followed by drying in a vacuum oven until constant weight to ensure the complete evaporation of the solvents. PVDF nanofibers were characterized through scanning electron microscopy (SEM) and X-ray diffraction (XRD) to determine morphology and crystalline structure, respectively.

### Sensor development, its embedding, and testing

**Figure 1:**
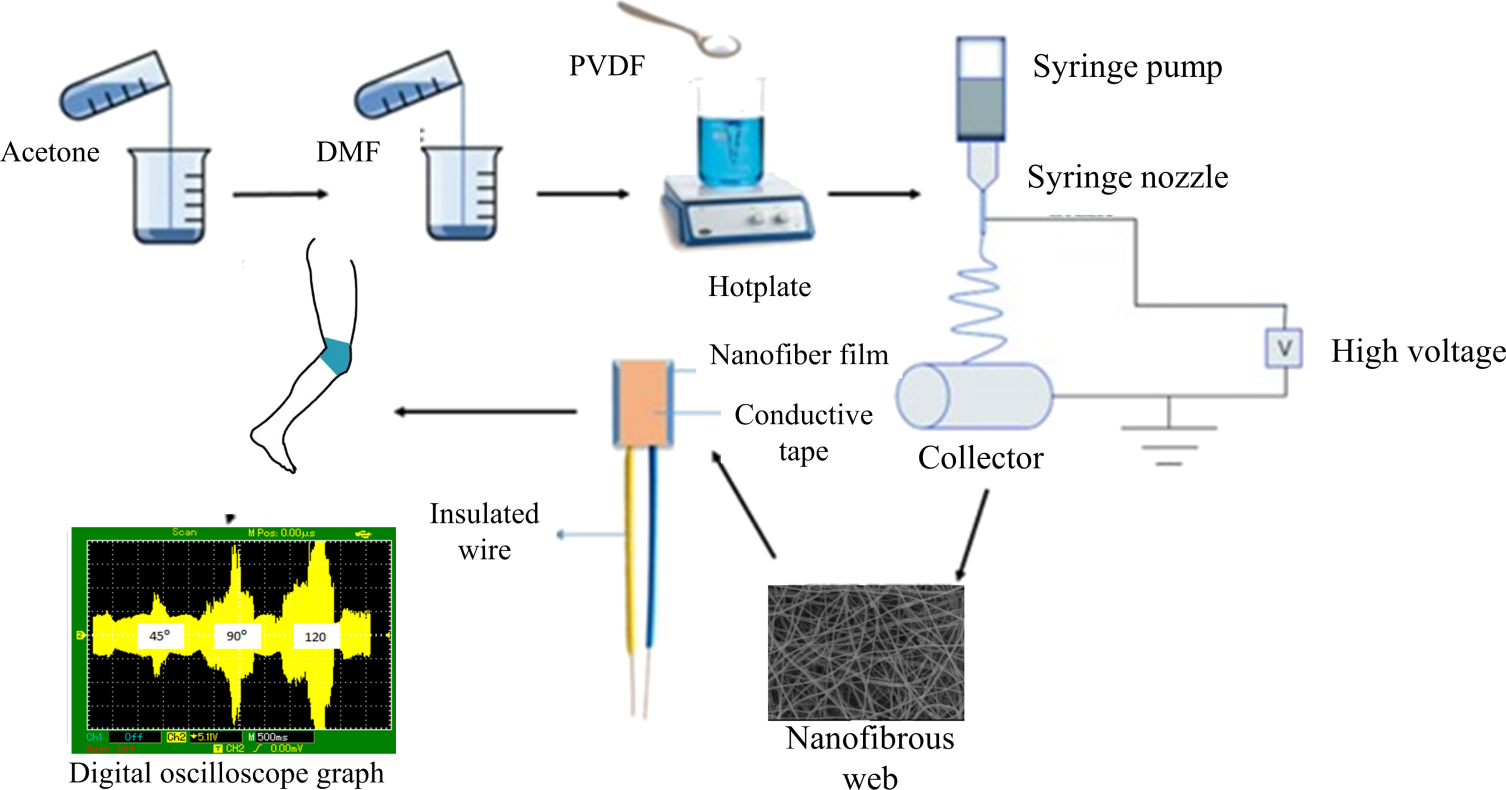
Illustration representing the scheme for sensor development.

The prepared PVDF nanofibrous mesh was folded into a square shape (4 cm^2^) with 2 mm thickness for sensor development. Subsequently, conductive tape was attached to both sides of the film in a way that it covered the maximum area of the sheet (Figure 1).

The developed piezoelectric sensor was tested in knee angle measurements using a digital oscilloscope. The sensor was exposed to low and high dynamic strains with varying frequencies to examine their effect on the output voltage. Then the developed sensor was integrated and stitched on a knitted fabric to check the effect of the bending angle on the output voltage. Staple spun polyester thread was used to stitch the sensor onto the knitted fabric by using a lockstitch machine. The sensor was worn on the knee and the bending angle of the knee was changed from 0° to 45°, 90°, and 120° to check the piezoelectric output with a digital oscilloscope.

## Results and Discussion

### SEM analysis

**Figure 2:**
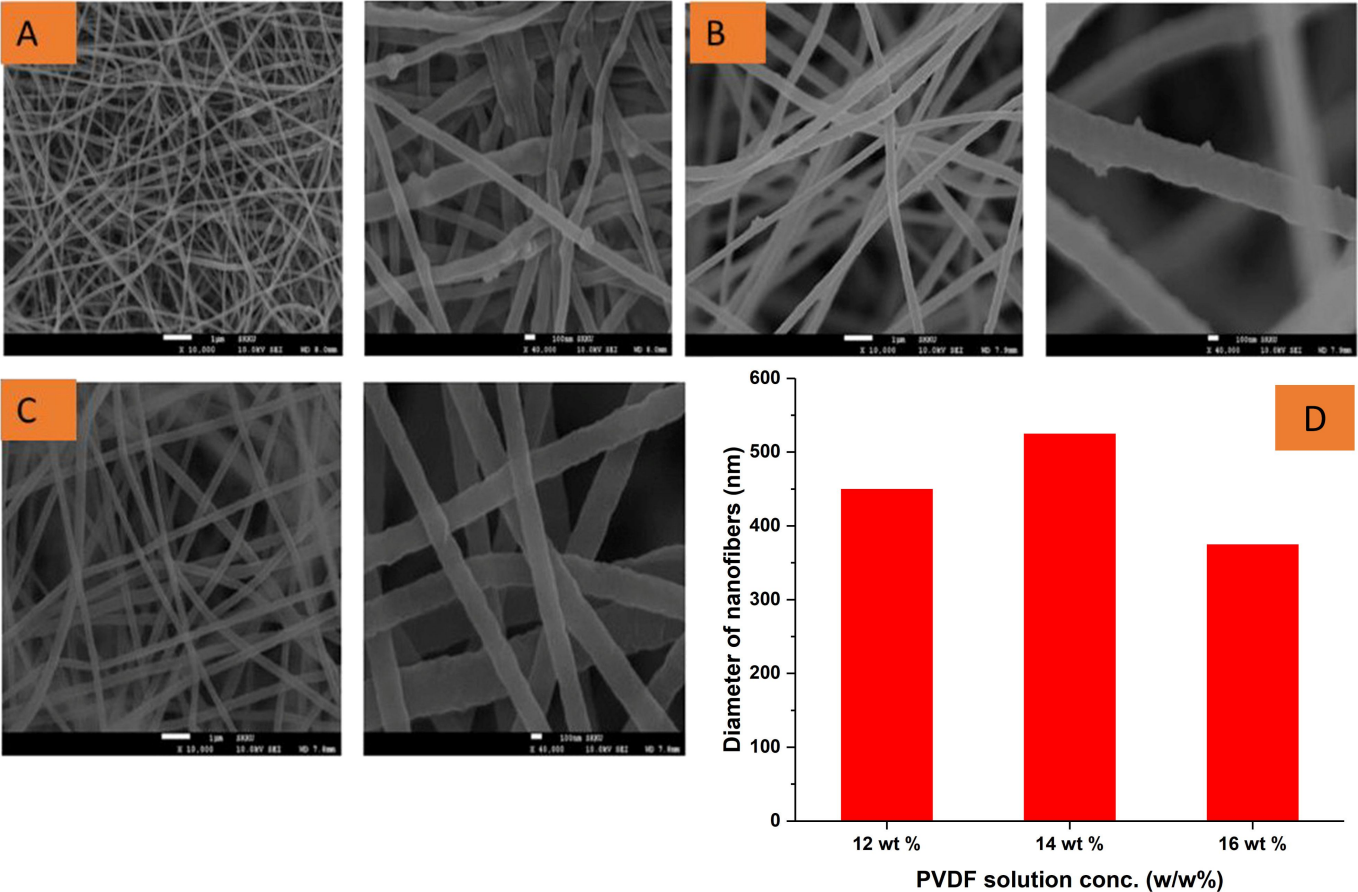
SEM images of nanofibers developed from 12 wt % (A), 14 wt % (B), and 16 wt % (C). PVDF solution and average diameter variation of nanofibers against different solution concentrations (D).

SEM was used to study the diameter and morphology of PVDF nanofibers developed through three different PVDF solutions (12, 14, and 16 wt %). The secondary electron images, taken at 10,000× and 40,000×, magnifications of prepared PVDF electrospun films from a 12 wt % polymer solution are shown in Figure 2A. The images show that the nanofibers have a more bead-on-string-like structure than the nanofibers obtained from 14 wt % solution (Figure 2B) due to incomplete solvent evaporation. The optimum polymer solution concentration is essential to obtain defect-free smooth fibers [14,37]. The nanofibers obtained from 16 wt % solution were smooth and presented a bead-free morphology (Figure 2C). Therefore, these fibers were selected for developing the piezoelectric sensor. The average diameter of the fibers obtained from different solutions is provided in Figure 2D.

### XRD analysis

**Figure 3:**
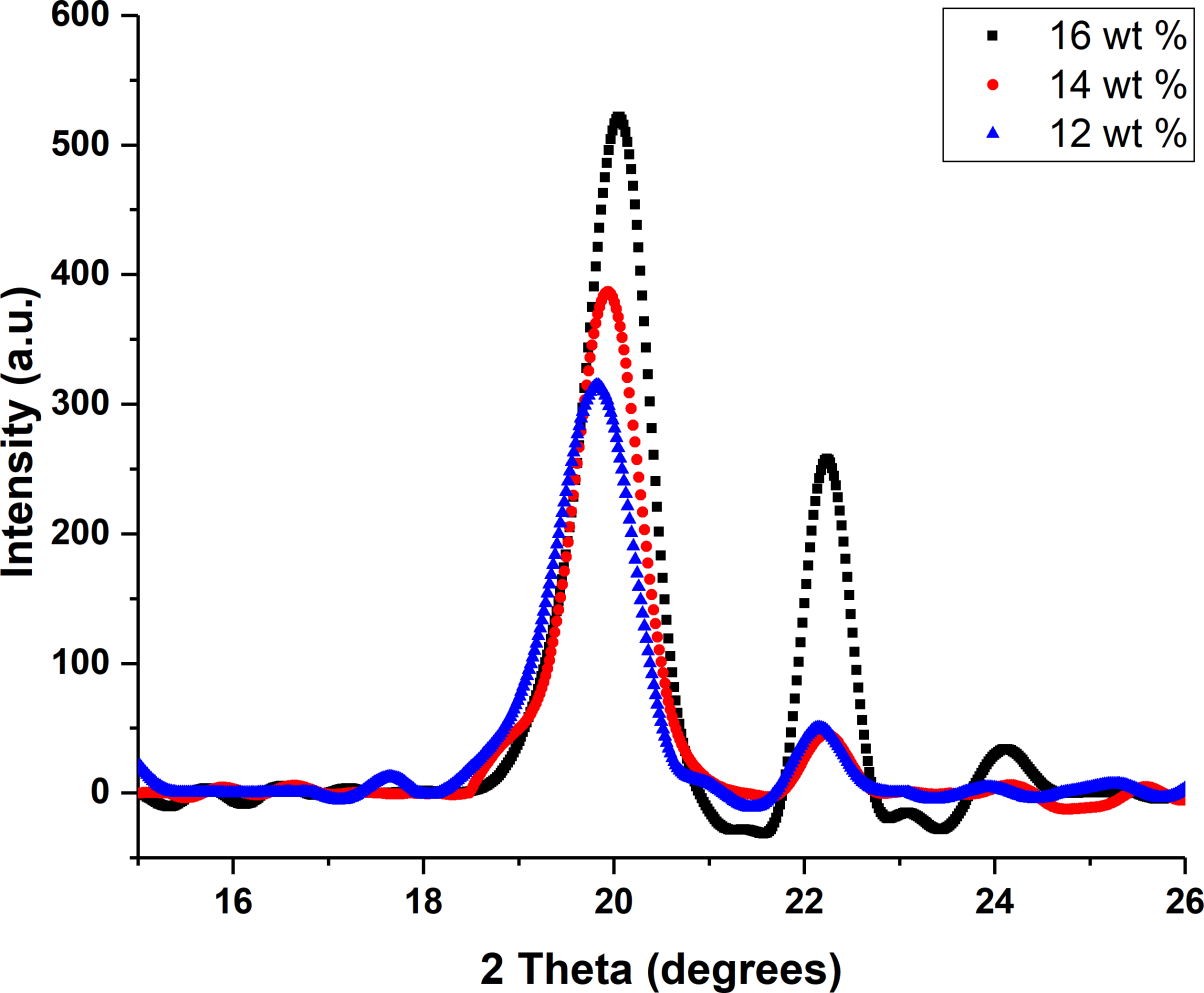
X-ray diffraction pattern of nanofibers at various concentrations.

PVDF exhibits four crystalline structures: α, β, γ, and δ [38]. Normally, all phases of PVDF show almost similar peaks with the exception of unique peaks that are used to identify the crystalline structure. X-ray diffractograms were used to analyze the crystalline structure [39] of prepared PVDF nanofibrous meshs obtained from different solutions to identify the beta phase in the fibers, which is primarily accountable for piezoelectric characteristics. PVDF showed its strongest peak near θ = 20°. The alpha phase has a peak around 18° [40], the γ phase has its intense peak at 19.2° [41], while the beta phase exhibits peaks between 20.04 and 22.03° (Figure 3).

It can be seen that the beta phase is dominant in the fibers obtained from the 16 wt % solution compared to the solutions of 12 and 14 wt %. The crystallinity was calculated from the XRD diffractograms and came out to be 52.3%, 54.6%, and 57.7% for the fibers made from 12, 14, and 16 wt % solutions, respectively. This phenomenon can be correlated with the high concentration of polymer solution. During evaporation of the solvent, polymer chains are more likely to form crystalline structures because they are closer together than in the solutions with lower concentration Also, when the polymer amount increases in the solution it increases generation capacity [37].

### Digital oscilloscope analysis

**Figure 4:**
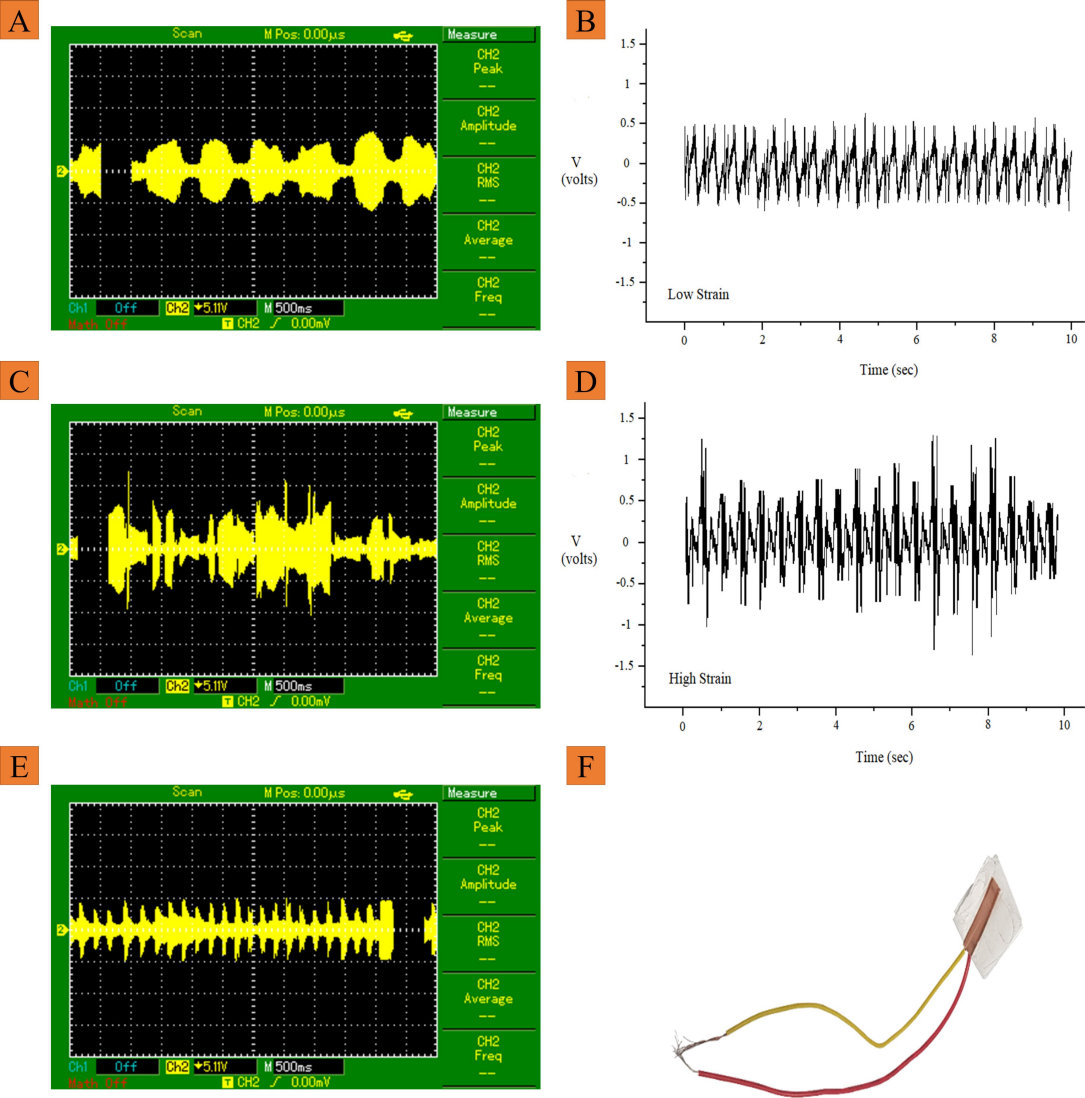
Digital oscilloscope graph of the sensor under low dynamic strain (A), the output voltage under low dynamic stress (B), digital oscilloscope graph of the sensor under high dynamic strain (C), the output voltage under high dynamic stress (D), digital oscilloscope graph of the sensor under low dynamic strain but with high frequency (E), and a piezoelectric sensor developed from 16 wt % solution (F).

The nanofibrous mesh made from the 16 wt % solution was selected for developing the sensor (Figure 4F). These nanofibers have a smooth and defect-free morphology with highly crystalline regions, which indicate the complete evaporation of the solvent and the presence of a large amount of polymer. To check the piezoelectric output of the developed sensor, a digital oscilloscope was used and the sensor was tapped with the fingers. Silicon gloves were used to avoid any possible static charges influence [42].

The sensor was placed on a smooth surface and the wires of the sensor were connected to the probes of a digital oscilloscope. Low dynamic strain was applied onto the sensor by pressing the sensor with a finger and then lifting the finger. The process was repeated continuously to obtain the effect of low strain on the piezoelectric output. Figure 4A illustrates the result of the digital oscilloscope showing output voltage when the sensor was under to low strain. The graph showing the piezoelectric output of the PVDF nanofibrous sensor under low dynamic strain is presented in Figure 4B. It shows that under low dynamic strain the energy generation is small. To check the impact of high strain, the previous process was repeated by pressing the sensor with a high dynamic strain. Figure 4C demonstrates the result of the digital oscilloscope showing output voltage when the sensor was under high dynamic strain. The result of the piezoelectric output of PVDF nanofibrous sensor under high dynamic strain is shown in Figure 4D. Under high dynamic strain, the generated energy is higher than under low dynamic strain, which shows the direct relation between pressure and piezoelectric output. To check the impact of the frequency on the piezoelectric output, the sensor wires were attached to the probes of the digital oscilloscope and a high-frequency dynamic strain was applied onto the sensor by pressing the sensor with a finger and lifting the finger at high speed. Figure 4E shows the result of the digital oscilloscope showing the output voltage of the sensor under dynamic strain at high frequency.

**Figure 5:**
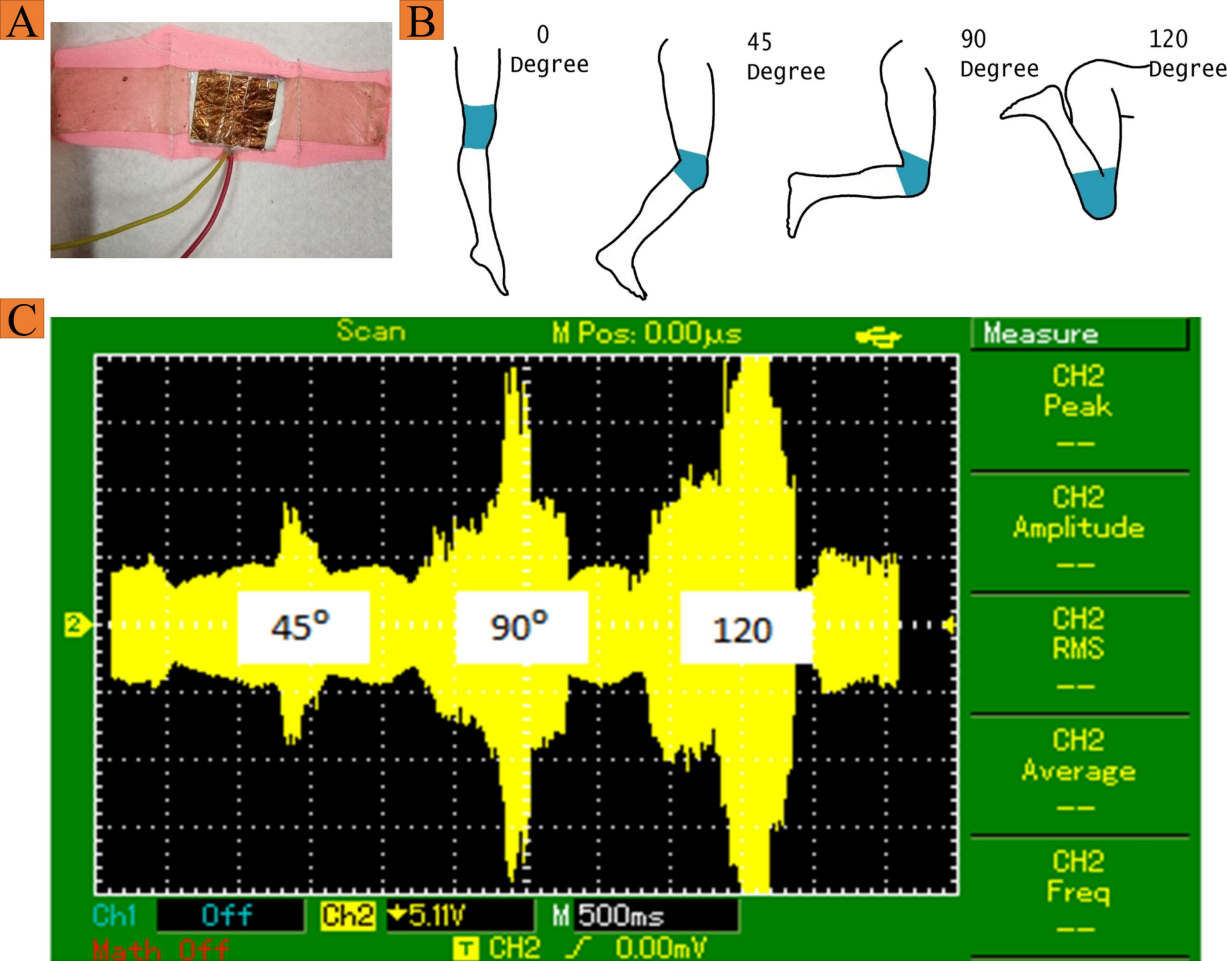
Integration of nanofibrous mesh into a knitted fabric for human body angle measurement (A), schematics showing the dressed knee at different bending angles (B), and digital oscilloscope graph at the corresponding angles (C).

To show the potential application of the developed sensor, the relation of the bending angle with the output voltage was established. For this, the sensor was integrated into a knitted fabric through stitching and a prototype representing a knee medical pad was developed (Figure 5A). The medical pad was worn onto a knee, and the knee was moved at different angles to check the output. The knee was moved from 0° degrees to 120° (Figure 5B). The effect of the bending angle on the output voltage was measured. When the bending angle was 45°, the output voltage was less than the output voltages at 90° and 120° because, with increasing angle, the sensor was more strained. Therefore, the bending angle and the output voltage are directly related. Figure 5C illustrates the voltage change with the change of the bending angle of the knee. The current density of the device has been calculated and compared with the current state of the art (Table 1). Clearly, the highest efficiency is shown by the device developed in the current study.

**Table 1:** Comparative study of the current density.

Sr. No.	Functional material	Form	Volts (V)	Current (nA)	Current density (nA/cm^2^)	Reference

1	PZT	nanotubes	1.00	40.00	—	[43]
2	PZT	single crystal	200.00	8000.00	150.00	[44]
3	PZT	composite	10.00	1300.00	0.20	[45]
4	PVDF-TrFE	thin film	7.00	58.00	0.56	[46]
5	PVDF	nanofiber	2.1	690	172.50	current study

## Conclusion

An experimental study on a textile-based piezoelectric sensor for human body angle monitoring has been performed. In this research, the polymeric material PVDF was used for the development of a piezoelectric nanofibrous sensor. SEM and XRD analyses were performed to determine morphology and crystalline phases of the developed nanofibers, respectively. The SEM analysis of nanofibers confirmed smooth, defect-free, and uniform fibers produced from a solution of high concentration (16 wt %). Additionally, the highest content of the beta phase was present in the nanofibrous mesh developed from the highly concentrated solution. Therefore, these fibers were selected for developing a piezoelectric sensor for subsequent studies. For this, the sensor was integrated into a knitted fabric through stitching to make a wearable textile-based piezoelectric sensor for human body angle monitoring. The piezoelectric output was measured by using a digital oscilloscope. The output voltage was high for high dynamic strain, which was also confirmed by changing the angle of the knee. The higher angle exerts more strain onto the sensor, which generates a high voltage in return. The piezoelectric output also increased when the frequency of the dynamic strain was increased. The textile-based piezoelectric sensor developed in this study has a great potential to be used as an angle measuring wearable device for the human body due to its high current density output and flexibility.
